# “Letter to the Editor: Talus Position Correlates With Dorsiflexion Range of Motion Following a Lateral Ankle Sprain: A Cross‐Sectional Study”

**DOI:** 10.1002/hsr2.70936

**Published:** 2025-06-22

**Authors:** Ahmad Furqan Anjum, Hammad Manzoor, Muhammad Abdullah

**Affiliations:** ^1^ Shaikh Khalifa Bin Zayed Al‐Nahyan Medical College Shaikh Zayed Postgraduate Medical Institute (SZPGMI) Lahore Pakistan

I want to applaud Toyooka et al. for their recent article “*Talus Position Correlates With Dorsiflexion Range of Motion Following a Lateral Ankle Sprain: A Cross‐Sectional Study*” in Health Science Reports [1]. This piece answers a clinically relevant and often underinvestigated question in the field of musculoskeletal rehabilitation—the biomechanical relationship between anterior talar displacement and limited ankle dorsiflexion after lateral ankle sprain. The authors should be praised for elucidating such a correlation using MRI measurements, adding an objective dimension to assessing post‐injury joint mechanics. Their thorough design and contribution are especially useful for clinicians and researchers interested in maximizing early intervention for recurrent ankle instability. This study sets a solid base for furthering the knowledge of ankle joint kinematics and addresses an important step toward customized rehabilitation. However, a few aspects of their study warrant closer examination before the conclusions drawn can be fully endorsed.

First, static MRI within the non‐weight‐bearing position measures anterior talar displacement. It does not account for dynamic joint mechanics under functional movement, such as load under dorsiflexion or gait. As restrictions of dorsiflexion usually occur under load, the correlation between talus position and ROM will be underestimated or misinterpreted. For example, Tavana et al. [2] point out that passive and active joint interactions cannot be differentiated using static MRI and that Digital Volume Correlation techniques with weight‐bearing MRI for real‐time strain analysis under joint loading should be used. Therefore, future studies should incorporate dynamic weight‐bearing MRI or 3D DVC techniques to measure talar position under functional tasks for greater ecological validity. Second, the study considers that dorsiflexion ROM depends only on the tibiotalar joint. Subtalar joint instability or limitations, nevertheless, may greatly influence the measurement of dorsiflexion. This simplifies joint biomechanics too much, perhaps pointing to limitations of motion indirectly through talar alignment at the tibiotalar joint. For example, Mittlmeier and Wichelhaus [3] proved that locking or instability of the subtalar joint changes the biomechanics of the foot and reproduces or enhances restrictions of dorsiflexion in the ankle. Therefore, the subsequent studies should incorporate subtalar joint examination based on either clinical or imaging techniques, like subtalar tilt or stress radiography, for the separation of actual limitations of dorsiflexion in the ankle joint. Third, although the study describes how WBLT was measured on both sides and how WBLT differences (WBLTD) were determined, it doesn't report any control of, or standardization for, foot position (pronation/supination), tibial rotation, or height of the arch during the test. There should also be no mention of whether the second toe, heel, or tibial tuberosity was aligned with a plumb line, wall, or floor markings—a usual method of controlling tibial position. It could artificially enhance or decrease WBLTD, impacting correlation strength with talar positioning. For example, Abdeen [4] pointed out that external tibial torsion or midfoot hypermobility may confound dorsiflexion ROM in weight‐bearing tests, resulting in changes unrelated to the ankle, hence altering. Therefore, future research should employ the foot posture index (FPI) or markers of motion capture to control for limb alignment during WBLT for improved measurement reliability. Fourth, the research assumes that the measure of dorsiflexion using the WBLT essentially captures primarily talocrural movement. However, new evidence indicates that WBLT includes midfoot and subtalar joint movement, in addition to the talocrural joint. This attributes restriction of movement, perhaps attenuating the correlation observed with talus anterior positioning. For instance, Smith et al. [5] demonstrated that the talocrural joint provides only ~62% of the movement detected by WBLT, and isolated interpretation becomes questionable. Therefore, future research should employ radiographic motion technology, or utilize motion capture equipment, to isolate talocrural movement from composite measures of WBLT. Fifth, the research fails to control for time variability since the lateral ankle sprain. Talar displacement and ROM will change over time from the acute to the chronic stages. This could lead to confounded correlation because late‐stage inflammation, joint laxity, and healing reactions vary by time since injury. Schurz et al. [6] emphasize that outcome variability has a close relationship with the chronicity of the injury and that time stratification by time since injury is necessary. Therefore, future research should stratify subjects into acute, subacute, and chronic stages, or account for time since injury as a covariate in models.

In conclusion, although Toyooka et al. have made a useful and clinically applicable examination of the correlation of talus position with dorsiflexion in the setting of lateral ankle sprain, there are some methodological considerations that deserve further clarification for the validity and relevance of their findings. Resolution of these other limitations—dynamic joint mechanics, subtalar impact, alignment standardization, composite joint contributions in the WBLT, and chronicity of the injury—will be necessary for subsequent research that further refines our knowledge of ankle biomechanics and optimizes focused rehabilitation techniques. Once again, I commend the authors for starting such a vital discussion.

## Author Contributions

**Ahmad Furqan Anjum:** conceptualization, writing – original draft, writing – review and editing, project administration, data curation, and supervision. **Hammad Manzoor:** conceptualization, writing – original draft, writing – review and editing, data curation. **Muhammad Abdullah:** conceptualization, writing – original draft, writing – review and editing, data curation.

## Conflicts of Interest

The authors declare no conflicts of interest.

## Transparency Statement

The lead author, Ahmad Furqan Anjum, affirms that this manuscript is an honest, accurate, and transparent account of the study being reported; that no important aspects of the study have been omitted; and that any discrepancies from the study as planned (and, if relevant, registered) have been explained.

## Data Availability

Data sharing not applicable to this article as no data sets were generated or analysed during the current study.
